# Fucosylated TLR4 mediates communication between mutualist fucotrophic microbiota and mammalian gut mucosa

**DOI:** 10.3389/fmed.2023.1070734

**Published:** 2023-03-16

**Authors:** Nanda N. Nanthakumar, Di Meng, David S. Newburg

**Affiliations:** Department of Pediatrics, Harvard Medical School and GI Unit, Massachusetts General Hospital, Boston, MA, United States

**Keywords:** Toll-like receptor 4, mutualist fucotropic bacteria, fut2 secretor gene, microbiota, fusosylated TLR4, intestinal mucosa and intestinal microbiota, cell signaling, transkingdom communication

## Abstract

**Objective:**

The glycans on the mucosa of suckling mice are predominantly sialylated; upon weaning, fucosylated glycans preponderate. This manifestation of mutualism between fucotrophic bacteria and the mature host utilizes a sentinel receptor in the intestinal mucosa; this receptor was isolated to distinguish its structural and functional features.

**Design:**

Provisional identification of the sentinel gut receptor as fuc-TLR4 was through colonization of germ-free mutant mice. Conventional mice whose microbiota was depleted with a cocktail of antibiotics were used to further define the nature and functions of fuc-TLR4 sentinel, and to define the role of the fucotrophic microbiota in gut homeostasis and recovery from insult. The nature of the sentinel was confirmed in cultured human HEL cells.

**Results:**

Fuc-TLR4 activity is distinct from that of TLR4. Activated mucosal fuc-TLR4 induces a fuc-TLR4 dependent non-inflammatory (ERK and JNK dependent, NF-κB independent) signaling cascade, initiating induction of fucosyltransferase 2 (secretor) gene transcription. *In vitro*, either defucosylation or TLR4 knockdown abrogates *FUT2* induction, indicating that fuc-TLR4 activity requires both the peptide and glycan moieties. *In vivo*, fucose-utilizing bacteria and fucose-binding ligands induce mucosal fucosylation. Activation of this pathway is essential for recovery from chemically induced mucosal injury *in vivo*.

**Conclusion:**

In mature mice, fucosyl-TLR4 mediated gut fucosylation creates a niche that supports the healthy fucose-dependent mutualism between the mammalian gut and its fucotrophic microbes. Such microbiota-induced Fuc-TLR4 signaling supports initial colonization of the secretor gut, recovery from dysbiosis, and restoration or preservation of intestinal homeostasis.

## Introduction

A popular supposition in the evolution of life is that the genesis of eukaryotes from a world filled with prokaryotes was accompanied by many successful eukaryotes participating in symbiotic mutualism with prokaryotes. In a mutualistic relationship, the specific pairing goes beyond providing essential nutrients to one another, and depends upon each mutualist binding to specific receptors in the other partner to mediate reciprocal interkingdom communication. Such mutualism between mammalian intestinal mucosa and the gut microbiota is of high relevance to human health.

The large and complex microbial ecosystem within the mammalian gastrointestinal tract had long been considered to be comprised of commensals, but increasing evidence indicates that the relationship is mutualistic (1–6). Both benefit. The mucosa provides a protected warm, wet, nutrient-rich niche for the microbiota. In return, harmonious cohabitation with mutualistic microbes maintains homeostasis of the intestinal mucosa (3, 4, 7, 8) and protects against various forms of insult. Mutualism involves reciprocal communication. Upon contact, microbes and host undergo mutually accommodating gene expression and signaling (4, 7, 9, 10). Most prior research on bacterial signaling in mammalian gut focused on activation of the inflammatory component of an immune response to pathogens, often mediated by toll-like receptors (TLRs).

Toll-like receptor 4 is activated by lipopolysaccharide (LPS), a cell wall component of Gram-negative bacteria, initially resulting in assembly of a signaling complex that includes MyD88. This complex activates a signal transduction cascade that enables nuclear translocation of transcription factors including AP1 and NF-κB (10–12), which activate transcription of pro-inflammatory genes to elicit IL-8 and TNF-α. The canonical signaling cascades activated by TLRs all lead to inflammation. Such inflammatory bacterial signaling by the innate immunity receptors TLR2 and TLR4 is also requisite for recovery from intestinal injury (13, 14).

In stark contrast to this paradigm, specific signaling pathways activated by pioneering species of fucotrophic bacteria during early colonization of adult murine gut mediate adaptive gene expression without activating inflammation (1, 9). Specifically, these microbes induce *fut*2 gene expression in the intestinal epithelium, leading to a highly fucosylated mucosa. The *fut*2 (secretor) gene encodes the galactoside 2-α-L-fucosyltransferase II (FucT II; EC 2.4.1.69), an inducible enzyme that adds α1,2 linked L-fucose to a terminal D-galactose of glycans. In the intestinal epithelial cell, these fucosylated glycans are generally transported to the extracellular glycocalyx of the intestinal mucosa (1, 9, 15). This fucosylated niche is hospitable to a microbial ecosystem that contains fucotrophic mutualists supportive of host resilience to a variety of mucosal insults (15–17). This *fut*2 expression is induced by early colonization *via* the ERK and JNK signaling pathways without stimulating the inflammatory NF-κB pathway (9).

In conventional mice, fully colonized post-weaning gut is heavily fucosylated, whereas colonized suckling gut exhibits a preponderance of sialylated glycans, with distinctly low fucosylation (17–19). The crossover at weaning suggested four potential regulatory mechanisms: (1) change in diet; (2) innate timing by developmentally sensitive genes; (3) modified hormonal milieu; or (4) a shift in microbiota. These possibilities were differentiated in germ-free mice, where the shift in fucosylation did not occur at weaning, eliminating change in diet, innate developmental gene control, and hormonal shifts as candidates (9). This conclusion is reinforced by the converse experiment, in which adult germ-free mice, whose gut mucosa are predominantly sialylated, upon being colonized by normal adult murine microbiota, initiate fucosylation of their mucosa. Moreover, when conventional colonized mice are treated with a cocktail of broad-spectrum antibiotics, their microbiota become depleted and dysbiotic, and the fucosylation of their gut reverts to the immature, sialylated state (9, 15). When such germ-free or antibiotic-treated bacterially-depleted mice are exposed to conventional fecal microbiota, mucosal fucosylation is rapidly induced. Furthermore, if germ-free or antibiotic-treated mice are inoculated solely with an individual fucose-utilizing bacterium species of normal mammalian microbiota, either *Bacteriodes thetaiotaomicron* or *B*. *fragilis,* mucosal fucosylation is rapidly induced (15, 20). This implies active regulation of gut fucosylation by normal intact microbiota and its major individual fucose-utilizing (fucotrophic) bacterial mutualists. Other systems also display a link between bacteria and *fut2* activation. For example, mucosal *fut2* expression is activated by IL-22 of lymphoid cells in a bacteria-dependent manner (21) and expression of epithelial IL-22 receptor enhances fucose-dependent host-microbe mutualism (22). Conversely, mucosal glycans of the distal gut, along with indigestible residual dietary glycans, modulate composition and function of the microbiota (23). Epithelial fucosylation regulates microbial metabolism and reduces bacterial virulent gene expression, thereby protecting the host (21, 24). These data are all consistent with interdependence between microbiota colonization and fucosylation of the intestinal mucosa (1–4, 7).

Mice whose microbiota has been disrupted by antibiotics and are unable to maintain intestinal fucosylation are also less able to recover from mucosal injury. Restoration of the microbiota re-establishes fucosylation and recovery from injury (9, 15). Colonization with only *B*. *fragilis* (9343), a fucotrophic gut bacterium, also fully reinstates fucosylation and recovery of homeostasis (15), whereas a mutant of *B*. *fragilis* (Δ*gmd-fcl*Δ*fkp*) that is unable to utilize fucose does not restore *fut*2 expression, mucosal fucosylation, or recovery of homeostasis. The concordance of fucosylation, colonization, and mucosal resilience implies a strong and active mutualism that underlies healthy homeostasis of the gut and recovery from insult. To prove this mutualism, the molecules mediating bidirectional interkingdom communication needed to be identified and defined. The studies described herein are focused on discovering and defining the molecule that mediates communication from mutualist fucotrophic bacteria in the murine gut to the nucleus of the intestinal mucosal cell.

Accordingly, this study addresses the following hypothesis: mature mammalian gut, when not colonized with fucotrophic bacteria at or after weaning, expresses a “sentinel” receptor molecule on the mucosal surface that, upon exposure to pioneering mutualistic fucotrophic bacteria, signals the intestinal epithelial cell nucleus and activates *fut2,* thereby inducing or renewing the accommodating fucosylated niche. Three postulates are tested: sentinel binding to fucotrophic bacteria induces *fut*2 expression through activation of an ERK and JNK non-inflammatory signaling cascade. The sentinel molecule has unique characteristics. Activation of this sentinel molecule is necessary and sufficient to promote fucosylation of the intestinal mucosa, thereby reinforcing intestinal colonization by fucotrophic mutualists and homeostasis of adult gut.

## Materials and methods

### Chemicals

Phenyl-β-D-galactoside, BSA, 2-mercaptoethanol, 2,4,6-t rinitrobenzene sulfonic acid (TNBS), and oxazalone (OXA) were from Sigma Chemical Co. (St. Louis, MO, United States). 10 mM GDP-[^14^C] Fucose (specific activity = 1.8 mCi/mmol) was from New England Nuclear Life Sciences (Boston, MA, United States). Taqman reverse transcription kits and enzymes were from Applied Biosystems (InVitrogen®, San Diego, CA, United States). Biotinylated and fluorescein-conjugated *Ulex europaeus* agglutinin-1 (UEA-I) were from E-Y Laboratories (San Mateo, CA, United States). Anti-Toll-like receptor-4 and anti-E-cadherin were from Santa Cruz Biotechnology (Santa Cruz, CA, United States). All other reagents were of analytical or molecular biology grade from Fisher Scientific (Fairlawn, NJ) or Sigma Chemical Co. Strains of *Bacteroides fragilis* were a gift from Dr. Laurie Comstock, Brigham and Women’s Hospital, Boston, MA, United States.

### Mice

C57B/6, C3H/Hej, C3H/Ouj, BALB/c mice, TLR4^−/−^, TLR2^−/−^, and MyD88^−/−^ mutant mice were from Jackson Labs, Bar Harbor, ME. Germ-free mice were maintained germ-free until immediately before being euthanized at 6 week of age. Ex-germ-free mice were produced by removing germ-free mice from their pristine environment at 4 or 6 weeks age and exposing them to conventional microbiota as a slurry of fresh fecal and cecal contents from age-matched conventional control mice (1 conventional mouse: 5 germ-free) through orogastric intubation, drinking water, and housing with conventional mice. All animals were euthanized between 12 and 3 PM (9). At sacrifice, mouse colons were harvested.

Mice were depleted of luminal bacteria by an antibiotic cocktail (100 μl antibiotic cocktail·mouse^−1^·day^−1^) in the drinking water for 2 weeks, whereupon commensal bacteria were introduced for 2 weeks, the mice were euthanized, and fucosyltransferase activity and *fut2* mRNA levels measured in each colon. The antibiotic cocktail contained Kanamycin (8 mg/ml), Gentamicin (0.7 mg/ml), Colistin (34,000 U/ml), Metronidazole (4.3 mg/ml), and Vancomycin (0.9 mg/ml). After the antibiotic cocktail was introduced, fresh fecal samples were collected daily from mouse and the presence of bacteria assessed in five different aerobic and anaerobic culture media (9, 15).

### DSS-treatment

Mice received 3.5% (wt/vol) DSS (40,000 kDa; ICN Biochemicals) in their drinking water *ad libitum* for 5 days, then ordinary drinking water (13, 15, 16). The amount of DSS water consumed per animal was similar across strains. Control mice received water only. For survival studies, mice were followed 12 days post start of DSS-treatment. Mice were weighed on alternate days, with % weight change calculated as: (weight at day x–day 0/ weight at day 0). Animals were monitored for rectal bleeding, diarrhea, and morbidity, including hunched posture and failure to groom. Similarly, consequences of 2.5% 2,4,6-trinitrobenzene sulfonic acid (TNBS) and oxazalone (OXA)-induced colitis (13, 15, 16) were compared to determine if phenomena observed in recovery from DSS could be generalized to recovery from mucosal injury *per se*, irrespective of cause. The three models produced essentially similar data, thus only DSS data are shown. Care of mice followed institutional guidelines under a protocol approved by the Institutional Animal Care Committee at the Massachusetts General Hospital and Virginia Tech.

### Fucosyltransferase activity

The entire small intestine and colon was removed and thoroughly flushed with ice-cold 0.9% NaCl. Fucosyltransferase activitiy was measured in samples of colon as described (9, 15). A 10% mucosal homogenate was prepared from colon in 0.1 M Tris–HCl buffer (pH 7.4), and the homogenate was centrifuged at 1,000 × *g* for 15 min to remove nuclei and cellular debris. The supernatant was then centrifuged at 105,000 × *g* for 1 h, resulting in a membrane fraction and soluble cell fluid. The resulting pellets were re-suspended in homogenization buffer (0.1 M Tris–HCl, pH 7.4), frozen as aliquots at −80°C, and used for the fucosyltransferase assay.

α1,2/3-Fucosyltransferase enzyme activity was assessed using phenyl-β-D-galactoside as the acceptor (9, 15). The reaction mixture for each assay contained, in a total volume of 0.1 ml, 25 mM phenyl-β-D- galactoside, 20 mM sodium phosphate buffer (pH 6.1), 10 mM fucose, 5 mM ATP, 20 mM MgCl_2_, 50 mM NaCl, 0.5% Triton X-100, 10 nmol GDP-[^14^C]fucose (0.1 μCi, specific activity 11 mCi/mmol; New England Nuclear), and homogenate containing 50–100 μg protein. GDP-fucose concentration was at saturation, and product formation was linear for 2 h of incubation for up to 100 μg of enzyme protein at 37°C. After 2 h, the reaction was terminated by addition of 100 μl of ethanol and dilution with 1 ml of 4°C H_2_O followed by centrifugation at 15,000 × *g* for 5 min. The supernatant was applied to C-18 Bond Elute cartridges (500 mg) that had previously been washed with 6 ml of acetonitrile followed by 6 ml water. After application of the sample, the cartridges were washed with 5 ml water to remove the radiolabeled precursor. The product, [^14^C]-fucosylphenyl-β-D-galactoside, was eluted with 1.5 ml 50% acetonitrile directly into scintillation vials. Five milliliters of scintillation cocktail (Ready Safe, Beckman, Fullerton, CA, United States) was added to each vial, and radioactivity determined by scintillation counting of the clear solution. Specific activity is expressed as nmol [^14^C] fucose incorporated/h/mg protein.

### Cell cultures

Human erythroleukemia cells (HEL cells), HeLa, and T84 (ATCC, Manassas, VA, United States) were grown in Dulbecco’s modified eagle medium (DMEM) supplemented with 10% FBS, 1% nonessential amino acids, 50 IU/ml penicillin, 50 μg/ml streptomycin, and 1% HEPES buffer. Cells were grown at 37°C in 95% O_2_ and 5% CO_2_. Cells were stimulated with LPS (1–100 ng/ml) and UEA1 (0.1–1 μg/ml); total RNA was isolated after 16 h and analyzed for mRNA by RT-PCR (9, 15). Media were collected for IL-8 quantification.

### IL-8 ELISA

Microtiter plates (96 well; Nunc-Maxisorp, Fisher Scientific, Pittsburgh, PA, United States) were coated overnight with anti-human IL-8 (R&D Systems, Minneapolis, MN, United States), and incubated for 70 min at 37°C with 100 μl of cell supernatant. After sequential incubations with rabbit anti-human IL-8 (Endogen, Woburn, MA, United States) followed by horseradish peroxidase (HRP)-conjugated goat anti-rabbit IgG (Biosource, Camarillo, CA, United States) and 2,2′ azino-bis (3-ethylbenz-thiazoline-6-sulfonic acid; ABTS), absorbance was measured at 405 nm. IL-8 concentrations determined from a standard curve of purified recombinant human IL-8 (R&D Systems, Minneapolis, MN, United States) and normalized to total cellular protein.

### Protein determination

Protein was measured by bicinchoninic acid binding (Pierce, Rockford, IL, United States) according to the manufacturer’s protocol, but modified for 96-well microtiter plates. Each protein sample (10 μl), was added to 200 μl working reagent and incubated at 37°C for 30 min. Absorbance at 560 nm was measured on a microtiter reader (BT 2000 Microkinetics Reader Spectrophotometer, Fisher Biotech, Pittsburgh, PA, United States). Concentration was calculated from a standard curve of bovine serum albumin (25, 26).

### Lectin histochemistry with UEA-1

Expression of fucosyl glycoconjugates on the mucosal surface was measured on frozen tissue sections using FITC-conjugated *Ulex europaeus* agglutinin-1 (UEA-1; Vector Laboratories, Burlingame, CA, United States). The middle 1 cm of the colon was fixed for 4 h at 4°C in 4% paraformaldehyde, washed in ice-cold PBS containing 30% sucrose overnight at 4°C, and embedded in optimal cutting temperature compound. Frozen sections (6–7 μm thick) were blocked with PBS containing 2% BSA and then stained with labeled lectin for 1 h (10 μg/ml). Sections were then washed three times in ice-cold PBS, mounted using Anti-Fade (Vector Labs), and analyzed by confocal microscopy (9).

### SDS PAGE analysis

Protein samples (30 or 50 μg) mixed with SDS sample buffer were loaded on 10–20% SDS Tris·HCl ready gels and transferred to Immun-Blot polyvinylidene difluoride membranes (Bio-Rad, Hercules, PA, United States). The membranes were blocked in blot A, 5% (wt/vol) Carnation nonfat dry milk (Nestlé, Solon, OH, United States) in Tris-buffered saline supplemented with 0.05% Tween 20 at room temperature for 1 h, then incubated overnight at 4°C with antibody or lectin. Blots were washed three times for 10 min each in blot A and then incubated with horseradish peroxidase-conjugated secondary antibody for 1 h at room temperature. After two 10-min washes in blot A and three 10-min washes in Tris-buffered saline, blots were developed *via* enhanced chemiluminescence (Supersignal; Pierce) (9, 25, 26).

### Total RNA isolation and quantitative RT-PCR

The RNA RNeasy Mini kit (Quigen, Valencia, CA, United States) was used to extract total RNA from homogenized tissue. RNA was reverse transcribed with random hexamers using a GeneAmp RNA PCR kit (Applied Biosystems, Foster City, CA, United States), and the cDNA was amplified using iQ SYBR Green Supermix (Bio-Rad) and 5 μM of each primer (25, 26). GAPDH primers were amplified in all samples. Duplicate cDNA samples were amplified 40 cycles for fut2, IL-8, and ICAM-1 and 42 cycles for fut1 for 1 min at 95°C and 1 min at 72°C. The threshold cycle (C_T_) was the cycle number at which fluorescence of the amplified product crossed a specified threshold value during exponential amplification. Mean Δ*C_T_* values of each mRNA were normalized by subtracting the mean C_T_ value of its control GAPDH mRNA. The primer sequences and calculations for relative quantification were carried out using the comparative *C_T_* method (2^−Δ*CT*^), as described (25, 26).

### Statistical analysis

The statistical significance of differences between treatment and control groups in all categorical comparisons was determined by factorial ANOVA, with results reported as mean ± SEM. Time to recovery was compared using Kaplan Meier survival analysis, with significant differences assessed by comparing the areas under the receiver operator curve (AUROC) by χ^2^. Statistical significance was set at α ≤ 0.05. Statistical analyses were performed using the XLStat software (Addinsoft, Brooklyn, NY).

## Results

### Colonization induces fucosylation of adult mouse gut mucosa

**Figure 1:**
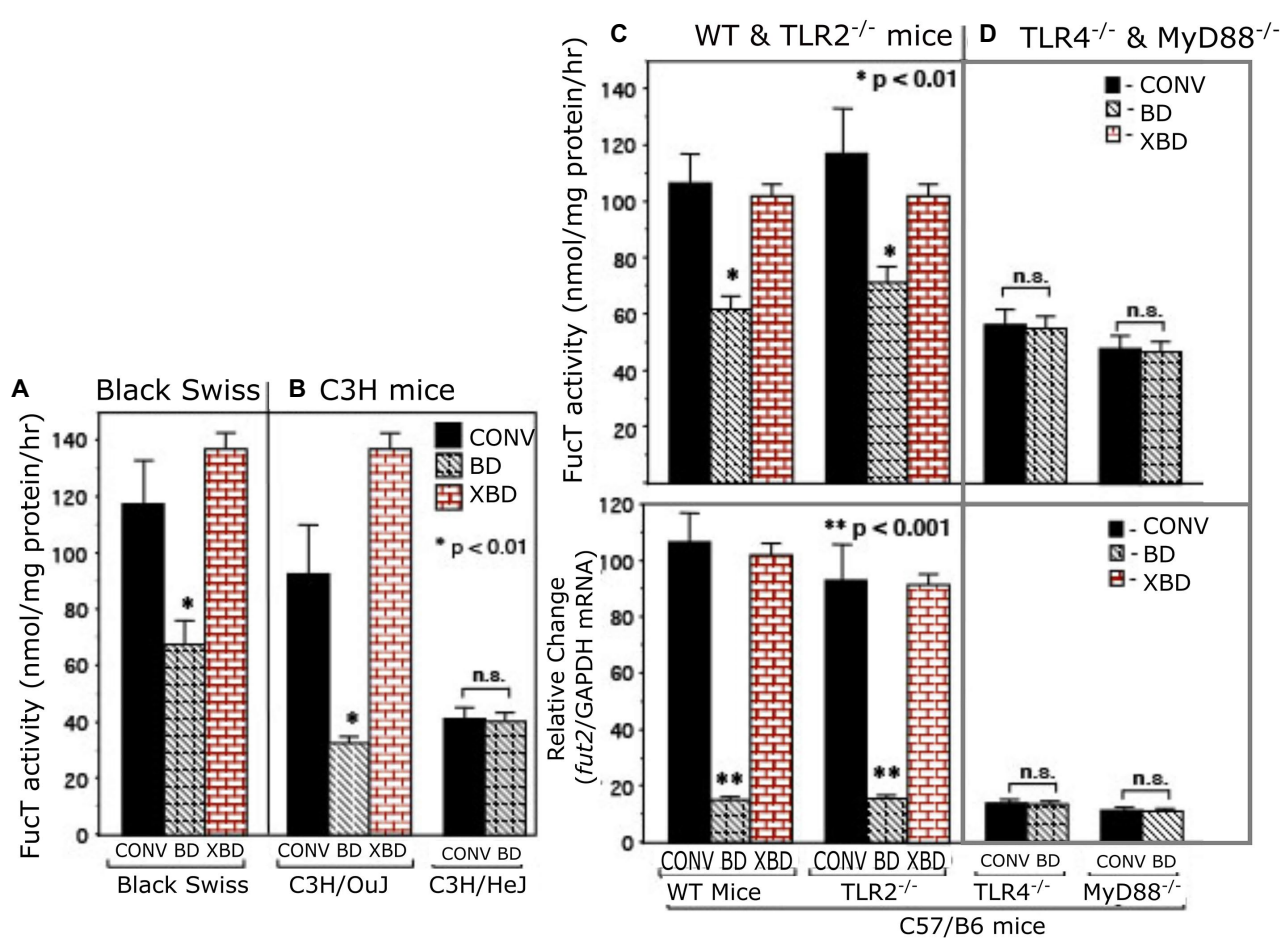
α1,2/3-fucosyltransferase (FucT) activity and fut2 mRNA in conventionally raised (CONV), bacteria-depleted (BD), and bacteria-repleted (XBD) mice. **(A)** Black Swiss, **(B)** C3H, **(C,D)** C57/B6. C3H/HeJ is isogenic strain of C3H/Ouj, but with an inactive TLR4 point mutation. The wild-type C57/B6 mice and TLR2^−/−^ exhibited normal colonization dependent induction of FucT activity and fut2 mRNA. TLR4^−/−^ and MyD88^−/−^ mutants displayed low expression FucT activity and fut2 mRNA, irrespective of colonization status. Thus, TLR4-mediated signaling is necessary for bacterial induction of fucosyltransferase expression. Data are mean ± SEM, *n* = 6–8 (*p < 0.01; **p < 0.001).

Depleting bacteria *via* an oral cocktail of antibiotics in 4–8-week-old mature mice decreased intestinal fucosylation and resulted in reversion to the predominantly sialylated mucosa typical of suckling gut (Figure 1 and supplement). Discontinuing the antibiotics and replenishing with adult mouse microbiota through exposure to the feces and bedding of conventional adult mice results in recovery of gut fucosylation, measured as increased α1,2/3-fucosyltransferase (FucT) activity (Figure 1A) and *fut*2 mRNA levels (Figure 1B). This occurred in all strains of mouse tested (Figures 1A–C), consistent with this aspect of mutualism being a general phenomenon.

### TLR4 is required for colonization-induced fucosylation

Toll-like receptors are known universal Gram-negative microbial sensors. Accordingly, TLRs were tested as potential mediators of bacteria-induced FucT activation and *fut2* mRNA expression in colonic mucosa (Figure 1). TLR4^−/−^, TLR2^−/−^, and MyD88^−/−^ mice (12, 13) were used to identify signaling pathways essential for colonization-dependent fucosylation. Neither TLR4^−/−^ nor MyD88^−/−^ mice exhibited the colonization-dependent fucosylation observed in the wild type, while TLR2^−/−^ mice did. Thus, both TLR4 and its downstream mediator, MyD88, are essential for bacteria-dependent up-regulation of *fut2* mRNA, FucT activity, and fucosylation of the mucosal surface, but TLR2 is not involved. Likewise, another strain of mouse, C3H/HeJ, is unable to express functional TLR4 (27), and also was unable to express *fut2* mRNA and FucT activity in response to colonization (Figure 1B), while its wild type C3H/OuJ could do so. Moreover, conventionally raised TLR4 mutant mice never achieved full fucosylation. These data are consistent with TLR4 activation being essential for initiation (and maintenance) of mucosal fucosylation, but whether TLR4 activation is sufficient was investigated next.

### In bacteria depleted mice, TLR4 stimulation induces fucosylation

**Figure 2:**
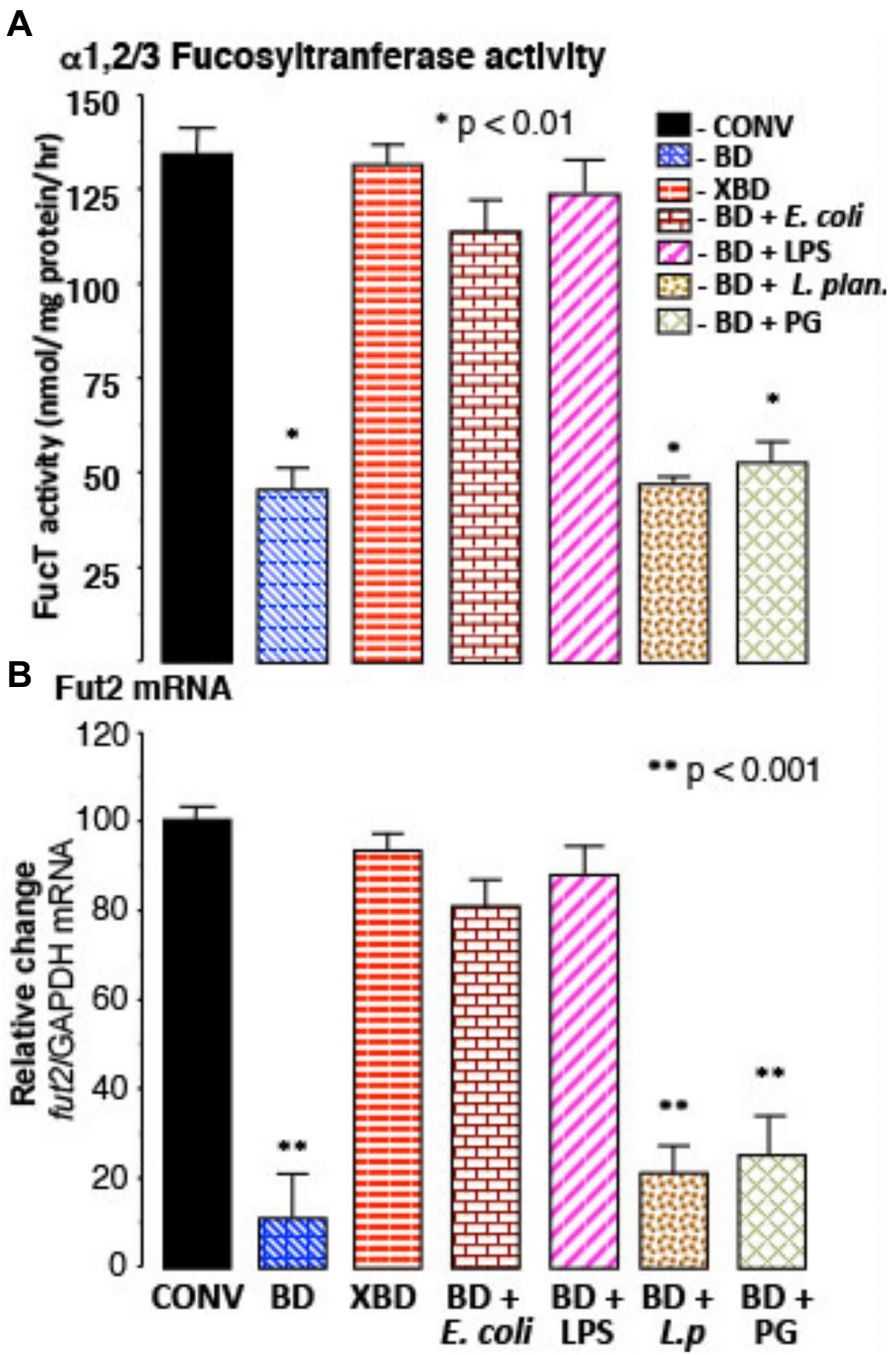
Recovery of FucT and *fut2* mRNA by recolonization. **(A)** α1,2/3-fucosyltransferase (FucT) activity and **(B)** fut2 mRNA in BD mice following recolonization with normal microflora (XBD), non-pathogenic Gram-negative *E*. *coli* (F18), or by ultra-pure LPS treatment. FucT activity and fut2 mRNA expression was not induced by Gram-positive bacteria *Lactobacillus plantarum* (*L*.*p*) or by peptidoglycan (PG) treatment. Thus, in bacteria depleted mice, ligands for TLR4, but not TLR2, induce FucT activity and fut2 mRNA. Data are mean ± SEM *n* = 8–10 (*p < 0.01; **p < 0.001).

In bacteria-depleted (BD) mice, orally administered TLR4 ligand, lipopolysaccharide (LPS), was able to induce *fut2* mRNA, FucT activity, and fucosylation (*p* < 0.01; Figure 2). Peptidoglycan (PG), the TLR2 ligand, did not. Germ-free mice manifest the same phenomena. Moreover, a commensal Gram-negative *E*. *coli* that expresses LPS on its surface also induced fucosylation (*p* < 0.01), while Gram-positive *Lactobacillus plantarum* (LP), which does not produce LPS, did not induce fucosylation in BD mice (Figure 2). Thus, luminal stimulation of TLR4 in BD mice is the critical signal necessary for colonization-mediated mucosal fucosylation. In all of these model systems, fucosylation is induced without activating inflammation, suggesting that TLR4 in BD mouse mucosa was different from prototypic TLR4.

### Non-colonized adult gut epithelium expresses a unique fucosylated TLR4

**Figure 3:**
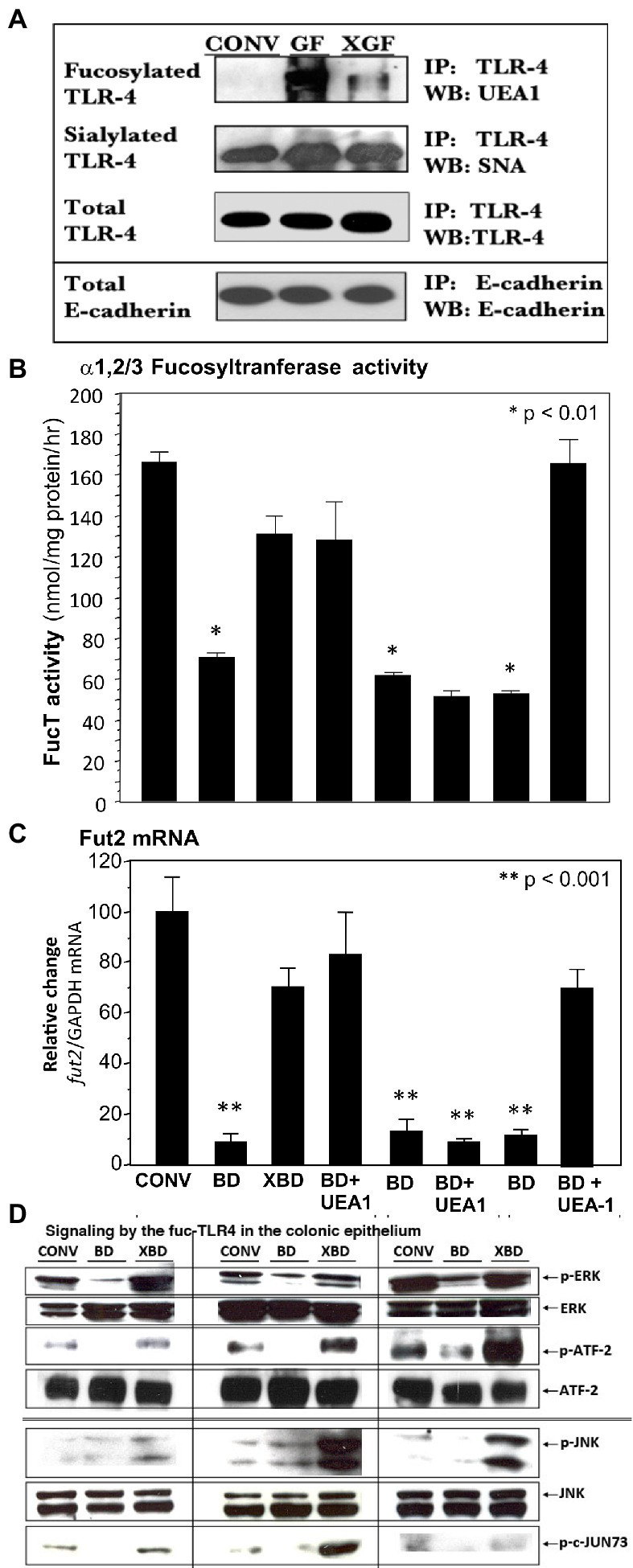
A novel fucosylated form of TLR-4 (fuc-TLR4) is present in intestinal epithelial cells of germfree mice **(A)**. UEA-I, which binds α1,2-fucosylglycans, did not bind the TLR4 of conventional (CONV) mice, but binds strongly to TLR4 of germ-free (GF) mice; colonization of germ-free (XGF) mice returns TLR4 to its non-fucosylated state. TLR4 sialylation, measured by SNA binding, was constitutive irrespective of colonization. E-cadherin a constitutive epithelial membrane protein, is used as the gel loading control; *n* = 6–8. UEA1 lectin binds α1,2-fucosylglycans. Feeding the fucose ligand UEA1 restored **(B)** α1,2/3-fucosyltransferase activity (FUT2) and **(C)** fut2 mRNA expression in bacteria-depleted (BD) mice, to levels equivalent to gut mucosa of colonized mice. This UEA1 induction does not occur in TLR4 knock-out mice, but does in TLR2 knock-outs. **(D)** When BD mice are recolonized (XBD), fed LPS, or fed UEA1, the increase in phosphorylation of ERK1/2 kinases and ATF2 (nuclear transcription factor 2) indicates activation of the ERK signaling pathway. Phosphorylation of JNK kinases and transcription factor c-Jun indicate activation of the JNK signaling pathway. This indicates that fucose binding *per se* is sufficient to induce recovery of the FucT activity and fut2 mRNA levels, that this activation requires TLR4, but not TLR2, dependent activation of ERK and JNK pathways. Data are mean ± SEM *n* = 6–8 (*p < 0.01; **p < 0.001).

The TLR4 from colonic epithelium of conventionally colonized (CONV), germ-free (GF) and newly recolonized germ-free (XGF) mice was isolated by immunoprecipitation (IP) and characterized by western blot (WB). The quantity of TLR4 protein (measured by binding to anti-TLR4 IgG_2a_) was similar for the three conditions, as was E-cadherin, a constitutive protein (Figure 3A). The TLR4s from all three conditions were sialylated, measured as *Sambucus nigra* agglutinin (SNA) lectin binding. In contrast, only the TLR4s from germ-free mouse colon bound *Ulex europaeus* agglutinin 1 (UEA1) lectin, indicating the presence of α1,2-linked fucosylation in addition to the sialylation. In the inverse experiment, protein from the three colonic epithelia was precipitated by UEA1 lectin, and only the precipitate from the germ-free (GF) mice was positive for TLR4 when probed by TLR4-specific IgG_2a_ antibody (Supplementary Figure 1A), confirming that only the germ-free TLR4 was fucosylated (fuc-TLR4). In other control experiments, the precipitate of an isotype specific control antibody, an IgG_2a_ that does not bind to TLR4, did not immuno-precipitate TLR4. Although fuc-TLR4 was quite evident in colonic epithelium, it was not found in other tissues, including peritoneal macrophages or liver of germ-free mice.

The expression of fucosylated TLR4 (fuc-TLR4) declined greatly after 2 weeks of colonization (XGF), and after 4 weeks, the TLR4 immuno-precipitate was indistinguishable from TLR4 from the gut of normal colonized mice. This led to the hypothesis that fuc-TLR4 could be a “sentinel” receptor on colonic epithelium, in which binding to its α1,2-linked fucosylated moiety of the fuc-TLR4 would induce fucosylation.

### UEA1 induces intestinal fucosylation

In bacteria-depleted BD mice, activation of TLR4 was sufficient to induce fucosylation, and the TLR4 was expressed as the unique glycoform of fuc-TLR4. The next hypothesis tested was that the fucose α1,2 linked moiety of the fuc-TLR4 is the essential feature of a sentinel receptor, and hence binding to the fucose moiety would be sufficient to initiate intestinal fucosylation. Bacteria-depleted mice were fed UEA1 while continuing antibiotic treatment to prevent confounding stimulation by re-colonization (Figures 3B,C). Feeding UEA1 to BD mice induced FucT activity (*p* < 0.01) and *fut2* mRNA expression (*p* < 0.001). Moreover, the level of fucosylation induced by only UEA1 was comparable to that induced by restitution of native mouse microbiota (Figures 2, 3B). As controls, other lectins that do not bind fucose, such as wheat germ agglutinin and *Maackia amurensis* agglutinin I, did not activate mucosal fucosylation, nor did fucose or fructosyloligosaccharides. Moreover, in antibiotic-treated TLR4^−/−^ and MyD88^−/−^ mutant BD mice, UEA1 did not induce colonic *fut2* mRNA (Figure 3B, 3rd from right), consistent with the inability of these mutants to express the fuc-TLR4 sentinel receptor, but UEA1 did induce fut2-TLR4 in antibiotic-treated BD TLR2^−/−^ mice (Figure 3B, right), whose TLR4 expression is intact. Taken together, these data strongly support the hypothesis that specific binding to the fucosylated glycan of fuc-TLR4 is sufficient to mimic the bacteria-induced fucosylation of the colonic mucosa. If fuc-TLR4 is the receptor that mediates fucosylation of BD mouse colon upon re-colonization, it should activate trans-cellular pathways that are essential to fut2 induction.

### Fuc-TLR4 signaling is mediated through ERK and JNK pathways

Re-colonization of BD mice requires ERK and JNK trans-cellular signaling pathways for induction of fucosylation; if activation of fuc-TLR4 is the requisite proximal event to induce fucosylation, feeding BD mice UEA1 and LPS should activate these same pathways. LPS and UEA1 induced intestinal ERK and JNK signaling intermediates at levels comparable to induction by re-colonization of XBD (Figure 3D); specific inhibitors of these signaling pathways significantly attenuated fuc-TLR4 mediated colonic fucosylation. Thus, fuc-TLR4 binding was both necessary and sufficient to specifically induce the same trans-cellular signaling of *fut2* induction through the ERK and JNK signaling pathways as are activated by re-colonization. However, feeding UEA1 *in vivo* does not provide direct evidence that UEA1 is binding specifically to the fuc-TLR4 molecule; this was tested by mechanistic studies in cell culture models.

### UEA-1 and LPS induce FUT2 in HEL and HeLa cells expressing fucosylated-TLR4

**Figure 4:**
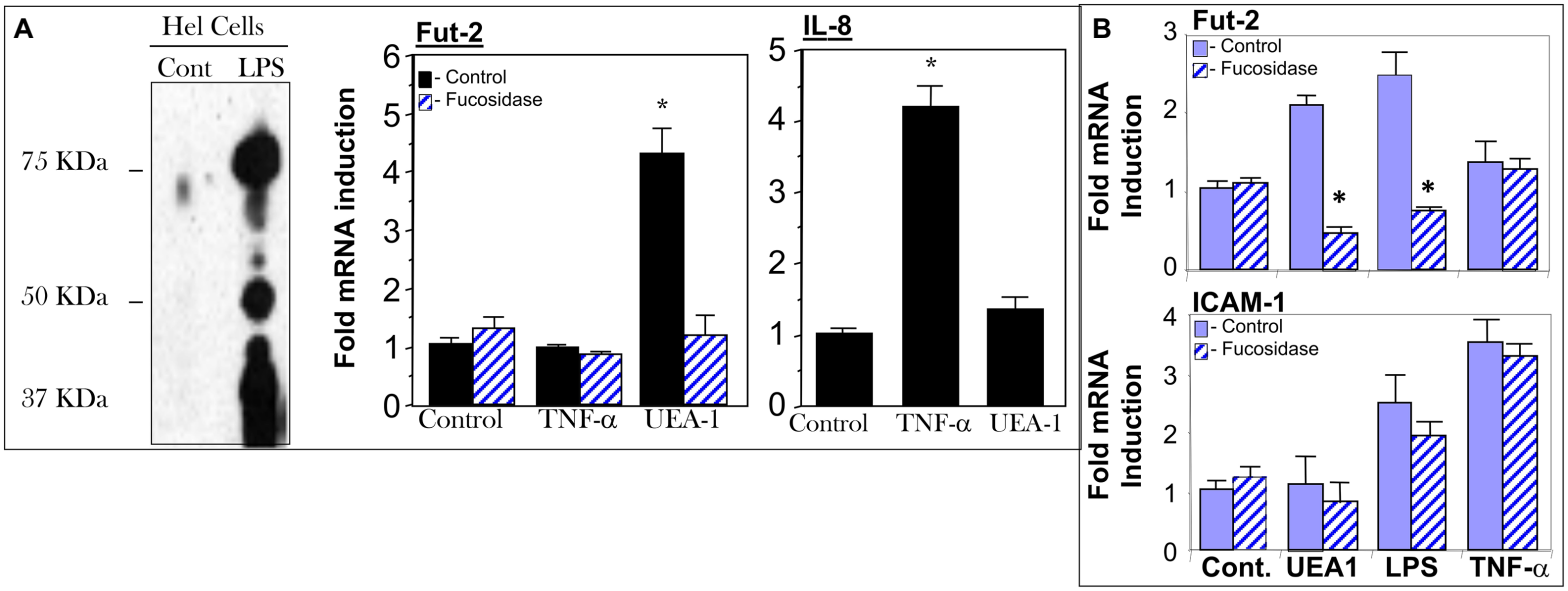
Cell models for testing UEA1 activation of fucosylated TLR4 (fuc-TLR4). Fuc-TLR4 is expressed constitutively in the HEL cell line derived from human erythroleukemia **(A)**, and HeLa cells tranfected with the TLR4 gene fucosylate the protein to form fuc-TLR4 **(B)**. Both of these models recapitulated the phenomena observed in intestinal mucosa of uncolonized mouse gut. Stimulation of HEL cells **(A)** or tranfected HeLa cells by fut-TLR4 ligands UEA1 or LPS induces cell surface fucosylation. UEA1 stimulated induction of fut2 mRNA was abolished in both models by prior removal of fucose from fuc-TLR4 by fucosidase. Stimulation of fuc-TLR4 by UEA1 does not stimulate proinflammatory pathways that lead to IL-8 production **(A)** or ICAM-1 production **(B)**; TNF-α is the positive control. UEA1, ligands specific for fucose or TLR4 induce fut2 mRNA, and consequent elevated fucosylation of the cell surface; the fucose specific ligand does not elicit an inflammatory response. Data are mean ± SEM *n* = 5–7 (*p < 0.01; **p < 0.001).

The HEL cell, a human erythroleukemia cell line, constitutively expresses fucosylated TLR4, and most of the remaining cell surface proteins are not usually fucosylated, providing a model to study fuc-TLR4 activation. HeLa cells transfected with the TLR4 gene also produce fucosylated TLR4. When HEL or TLR4 transfected HeLa cells were treated with UEA1 or LPS, within 16 h the other membrane proteins became highly fucosylated (Figures 4A,B), indicating that fuc-TLR4, when activated by its ligands, induces α1,2 fucosytransferase gene expression in these human cell models. The ability of UEA1 to induce FUT2 mRNA and to activate membrane fucosylation is consistent with UEA1 activating fuc-TLR4. Moreover, treating cells with an α1,2-specific fucosidase to remove the fucose from fuc-TLR4 abrogated the ability of UEA1 to induce *fut2* mRNA expression (Figure 4A). Both UEA1 and LPS induced *fut2* mRNA in a dose dependent manner (*p* < 0.01), and did not induce *fut1* mRNA (*p* > 0.4; Supplementary Figure 2). The concentration of LPS needed for inducing FUT2 expression in HEL cells is two orders of magnitude greater than that required for inducing IL-8 expression *via* non-fucosylated TLR4. This indicates involvement of TLR4 in FUT2 induction, but no involvement in this signaling at typical levels of LPS. Conversely, T84 or CaCo-2 (colonic epithelial) cells (16, 17) do not produce endogenous fuc-TLR4, and neither LPS nor UEA1 is able to induce FUT2 expression or cell-surface fucosylation in these cells. TNF-α induced release of IL-8 in HEL cells and release of ICAM-1 in HeLa cells (*p* < 0.01), indicating that inflammatory signaling pathways are intact in these cell lines. In these cells, UEA1 activates the fuc-TLR4 dependent fucosylation without inducing inflammation, indicated by the lack of concomitant increases in IL-8 or ICAM-1 release (*p* > 0.3; Figure 4). These models are concordant with the response of bacteria depleted mice to UEA1. Thus, fucosylation of TLR4 is necessary for UEA1 to induce *fut2* mRNA levels in human cells *in vitro*, as it is in mice *in vivo*. This conclusion could be ascertained by direct evidence that the fucosylated molecule activated by UEA1 was TLR4.

### UEA1- and LPS-induced fucosylation of HEL cells requires TLR4 expression

**Figure 5:**
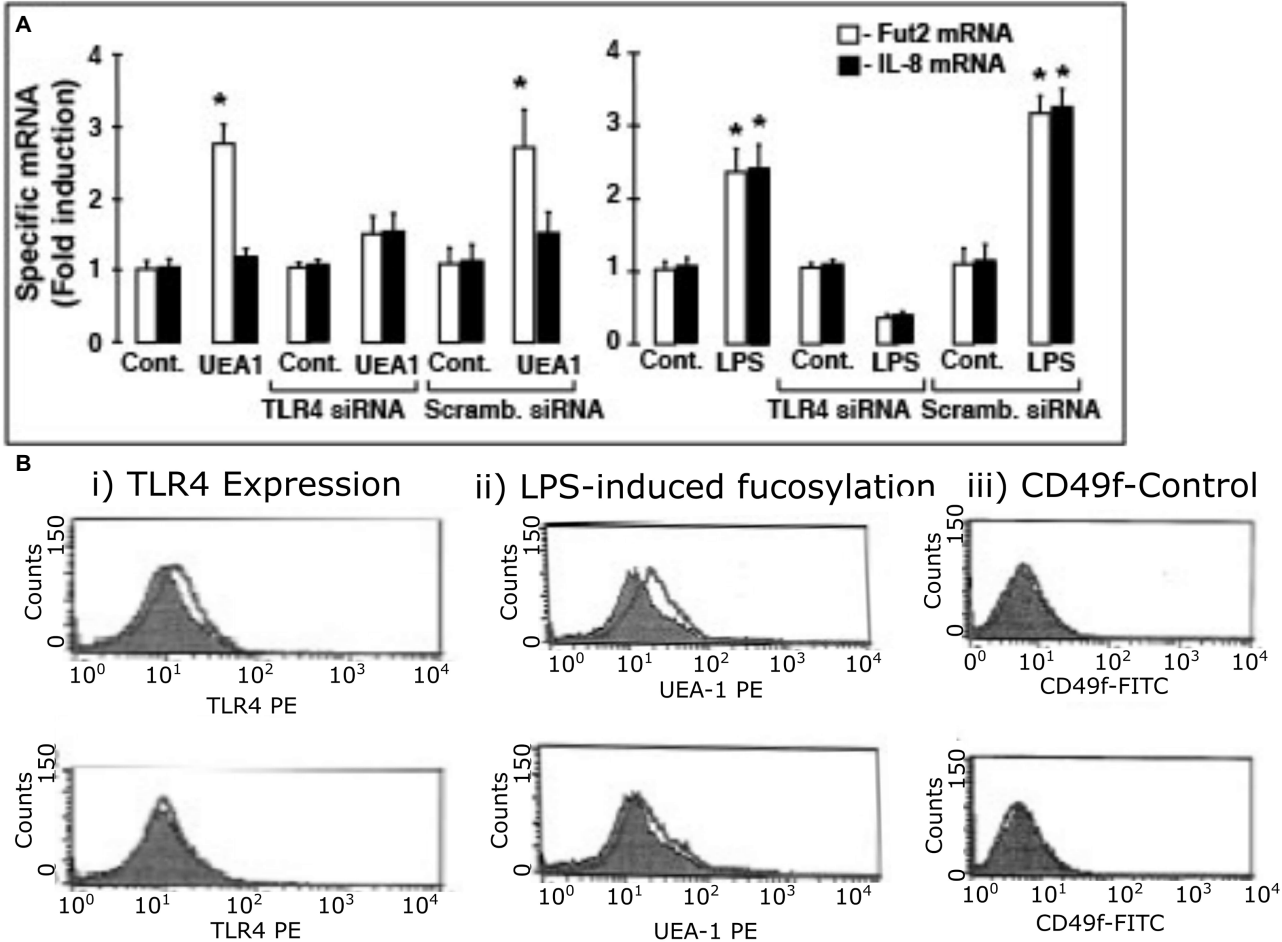
TLR4 expression is essential for induction of fut2 mRNA expression and cell surface fucosylation by UEA1 and LPS. To test whether TLR4 expression is *sine qua non* for Hel cell induction of fut2 mRNA, the consequence of TLR4 knock-down by siRNA on response to the TLR4 ligands was measured **(A)**. The induction of FUT2 mRNA transcription by LPS (right) or by UEA1 (left) is inhibited by a TLR4 specific siRNA, but not by a scrambled siRNA. In contrast, only induction of IL-8 transcription by LPS was affected by the TLR4 siRNA, as the UEA1 did not induce IL-8. The induced cell surface fucosylation, measured by FACS analysis, followed the same pattern **(B)**, consistent with TLR4 mediation of FUT2 mRNA induction regulating cell surface fucosylation; CD49 was the constitutive control. Thus, activation of fucosylated TLR-4 is necessary for inducing fut2 mRNA and cellular fucosylation. Data are mean ± SEM *n* = 4 (*p < 0.01; **p < 0.001).

To confirm that fuc-TLR4 *per se* mediates induction of *fut2* mRNA transcription by UEA1, the expression of TLR4 in HEL cells was knocked down with siRNA (Figure 5). The efficiency of TLR4 knockdown was measured as reduction in TLR4 mRNA levels (Supplementary Figure 3) and in reduced cell surface expression of TLR4 as measured by FACS analysis (Figure 5B; Supplementary Figure 4). Control cells treated with scrambled siRNA remained sensitive to UEA1 induction of fut2 mRNA expression and cell surface fucosylation without the activation of IL-8 mRNA production, and toward LPS eliciting all three. However, after siRNA knockdown of TLR4 expression, UEA1 no longer induced fut2-mRNA and cell surface fucosylation. Likewise, LPS no longer induced fut2-mRNA, cell surface fucosylation, or IL-8-mRNA expression (Figures 5A,B). These data confirm that TLR4 is required for induction of *fut2* mRNA and its downstream sequellae; that its fucosylation is essential is verified in Figure 4. Thus, these HEL cell studies provide independent confirmation that UEA1 induction of *fut2* mRNA transcription and surface fucosylation is mediated through fuc-TLR4. The next question addressed whether fuc-TLR4 signaling has a functional role in maintaining mucosal homeostasis.

### Fuc-TLR4 signaling is necessary for recovery from mucosal injury during dysbiosis

**Figure 6:**
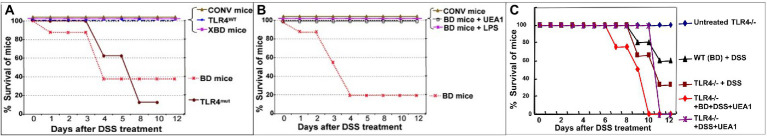
Fuc-TLR4 signaling is indispensable for intestinal homeostasis and recovery from injury provided by gut microbes. Conventionally colonized mice recover from the chemically induced mucosal injury caused by ingestion of DSS **(A)**. In contrast, only a fraction of mice whose microbiota are depleted by a cocktail of antibiotics recover from DSS-induced injury (BD mice). BD mice recolonized by mouse microbiota recover from this mucosal injury. Almost all TLR4^−/−^ mutants are unable to recover from DSS mucosal injury, consistent with the essential nature of TLR4 mediating bacterial signaling needed for recovery and maintenance of mucosal integrity. BD mice that are treated with a fuc-TLR4 ligand, either UEA1 or LPS, also recover fully **(B)**. Thus, activation of fuc-TLR4 in the absence of recolonization also allows full recovery. Thus, fuc-TLR4 signaling is necessary and sufficient for recovery of intestinal homeostasis following DSS induced injury of the gut. Conversely, neither UEA1 nor LPS can rescue TLR4^−/−^ mice (nor MyD88^−/−^ mice, not shown) from the injury caused by DSS treatment **(C)**. The data are consistent with fuc-TLR4 signaling being the essential signaling agent for microbiota mediated mucosal restoration of homeostasis. (*n* = 15–20; *p < 0.01; **p < 0.001).

The functional role of fuc-TLR4 signaling in mucosal repair and recovery of homeostasis was investigated by inducing colitis in BD mice. Fully colonized conventional control mice were able to fully recover from chemically induced mucosal injury induced by 3.5% DSS, whereas the same insult in BD mice resulted in 60% mortality (Figure 6A). Restitution of typical mouse microbiota (XBD) allowed full recovery from DSS injury, as does treatment with a fuc-TLR4 ligand, either UEA1 or LPS (Figure 6B). Consistent with the *in vitro* model, the amount of LPS required to rescue the BD mice from colitis-induce mortality is two orders of magnitude higher than that needed for LPS-mediated sepsis in fully colonized mice. Lectins that do not bind fucose, such as wheat germ agglutinin and *Maackia amurensis* agglutinin I, did not activate mucosal fucosylation, nor restore the ability to recover from mucosal insult of BD mice. Thus, activation of fuc-TLR4 is the critical feature supporting a return to mucosal homeostasis in BD mice. The essential nature of fuc-TLR4 signaling was reinforced by the inability of UEA1, conventional microbiota, or their combination to reverse DSS-induced damage in TLR4^−/−^ mutants (Figure 6C) or MyD88^−/−^ mutants. Likewise, fuc-TLR4 signaling is also required for recovery from TNBS and OXA, which induce related forms of chemically-induced mucosal injury. Thus, fuc-TLR4 signaling is a critical component for recovery from injury in dysbiotic gut through restoration of mucosal homeostasis.

In aggregate, these data are consistent with the newly discovered fuc-TLR4 being a unique “sentinel” sensor molecule on the surface of intestinal mucosa of uncolonized mature gut. An initial inoculum of fucose utilizing bacteria activates fuc-TLR4, which induces fucosylation of the colon, creating a niche that fosters colonization by fucotrophic mutualists of the microbiota.

## Discussion

A reciprocated beneficial symbiotic relationship is defined as mutualism (2, 3, 28, 29). Mutualism is a widespread feature of life that includes bidirectional communication to facilitate harmonious coexistence. Dialogue between animals and their bacterial mutualists can be through direct receptor-mediated trans-cellular signaling, but this had not been defined for mammalian gut microbiota (2, 9). TLRs are the foremost microbial pattern recognition receptors of the vertebrate innate immune system found in the intestinal mucosa; they induce an inflammatory response that preserves life but causes collateral damage to the host (10–12). When mammalian gut microbes were considered commensal, this immune response was thought necessary for their confinement to the gut lumen. Mutualists, on the other hand, usually communicate with their hosts to initiate mutual adaptation without inducing detrimental inflammation (28, 29). Removal of the symbiont typically results in reversion to the pre-mutualistic state.

The interactions of mice with their microbiota observed in this study are consistent with this general pattern of mutualist interkingdom communication. Consumption of the cocktail of broad-spectrum antibiotics caused reduction and disruption of mutualist microbiota, and was accompanied by reversion of the fucosylated intestinal mucosa to the sparsely fucosylated form. This was observed in all segments of the intestine, but the phenomenon was most pronounced in the colon, which exhibits the highest degree of colonization, the highest induction of signaling, and was the primary site of injury by chemically-induced colitis. Therefore, this study focused on the colon, where the robust appearance of the novel fucosylated isoform of TLR4, fuc-TLR4, during weaning or recolonization was accompanied by fucosylation of the mucosa. Stimulation of this fuc-TLR4 did not induce inflammation, even when exposed to typical physiologic levels of LPS, but activated a signaling cascade that resulted in copious fucosylation of the colonic mucosa. Thus, fuc-TLR4 could be functioning as a sentinel that restores fucosylation when the renewed opportunity for recolonizing with fucotrophic symbionts is detected. This system would allow expenditure of resources needed to maintain mutualism (fucosylation) only when there is the possibility of reciprocation. Saccharolytic species of gut microbiota engage in glycan foraging (1, 20, 30, 31), and major human bacterial mutualists grow best when their media contains fucosylated glycans, especially those containing the fucose α1,2 moiety (30). We propose that this heavily fucosylated mucosal niche induced by putative pioneering or principal species forms a base for a fucose-dependent heterotrophic microbial food web that underlies mutualist gut microbiota of mice and men. This is consistent with the observation that fucose-utilizing bacteroides are often the largest component of the adult mammalian gut microbiota (1, 2, 20, 23). The same mechanism observed at weaning in mice could likewise control the developmental transition to a fucotroph-dominated microbiota in humans. This fucose dependent mammalian microbial community promotes homeostasis of the intestinal mucosa and resilience to several forms of injury. Thus, the studies described herein support a central role of fuc-TLR4 as a putative sentinel molecule mediating the initiation of this strongly mutualistic relationship between humans and their fucotrophic microbiota. If so, this raises the possibility that analogous microbial food webs could exist around other sugars, such as sialic acid or galactose; these could be the base for alternate heterotrophic gut microbiota, potentially a sialic based microbiota in suckling mammals, and a galactose dominant microbiota in non-secretor humans. This seems a promising topic of further research.

This novel fucosylated form of TLR4 exhibits unique functional features that would allow detection of fucotrophic bacteria by the gut mucosa with activation of signaling pathways that lead to mucosal fucosylation without inducing inflammation. These changes persist until the gut becomes colonized, further supporting the role of fuc-TLR4 as a sentinel whose high levels attenuate toward conventional TLR4 levels upon restitution of the microbiota. Glycosylation of TLRs has precedent in other systems, where glycosylation can alter TLR expression, sub-cellular localization, and function. For example, glycosylation of TLR3 alters both subcellular trafficking and recognition of its ligand, single-stranded RNA (32). Likewise, specific point mutations in human TLR4 eliminate asparagine *N*-glycosylation sites, and the resulting alterations in glycosylation modify trafficking to the cell surface, formation of receptor complexes, and ligand-induced JNK dependent IL-8 transcription (33, 34). Different ligand affinity with differential glycosylation is consistent with the extreme differences observed herein between fuc-TLR4 and conventional non-fucosylated TLR4. Not only does fuc-TLR4 acquire the ability to be stimulated by fucose ligands, but activation of fuc-TLR4, even if by LPS, also results in non-inflammatory biological outcomes that are distinct from those of non-fucosylated TLR4.

**Figure 7:**
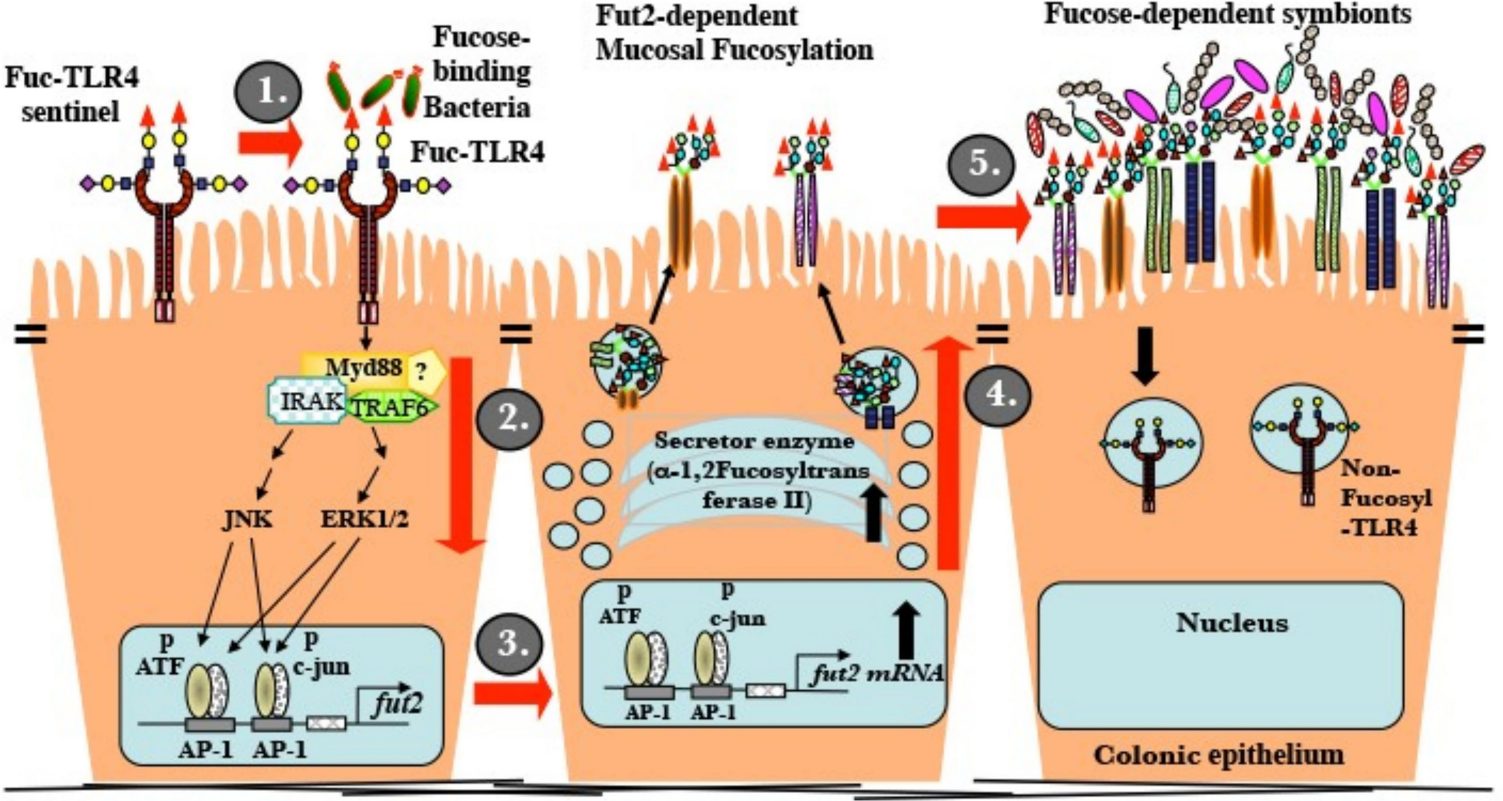
Mature uncolonized mouse gut expresses a unique fucosylated TLR4 (fuc-TLR4). (i) Fucose utilizing or Gram-negative bacteria bind and activate fuc-TLR4. (ii) The complex activates Myd88, ERK, and JNK dependent signaling that activates the AP-1 domain of the *fut2* gene. (iii) *Fut2*-mRNA is transcribed. (iv) FucT II is expressed and activated; glycans targeted to the apical glycocalyx are heavily fucosylated. (v) Luminal surface fucose serves as a base for a fucose-dependent heterotrophic microbial food web, the mutualistic human microbiota of mature secretors. A fully fucosylated and colonized gut resists insults to homeostasis.

The established ligand for standard non-fucosylated TLR4 is LPS. At low ng/mL levels, LPS activates inflammatory signaling mediated through NF-κB, but not mucosal fucosylation. Conventional TLR4 is insensitive to UEA-1 and other fucose ligands. In contrast, LPS induces the NF-κB cascade of fuc-TLR4 only at greater than 100 fold its usual threshold (low μg/mL, Supplementary Figure 2). Measurements of activation by UEA-1 supports the conclusion that binding to the fucosylated glycan moiety of fuc-TLR4 is functionally equivalent to activation by low ng/mL levels of LPS. In both *in vitro* models in this study, fuc-TLR4 binding does not activate the NF-κB cascade, but only the ERK and JNK pathways. This results in phosphorylation of transcription factors ATF-2 and c-jun. In the nucleus, phosphorylated ATF-2 and c-jun bind to AP1 promoters to initiate fut2 transcription (Figure 7). FucT II enzyme (alpha1,2-fucoslytransferase II) is translated, processed, and activated in the ER-Golgi complex, resulting in extensive α1,2-fucosylation of mucosal epithelial cell surface glycans (9). In contrast to other reported changes with alternate TLR glycosylation, fucosylation of TLR4 changed its fundamental function. The ability of a simple terminal α1,2-fucosylation to convert the TLR4 molecule from an innate immune signaling receptor that mounts pro-inflammatory defensive processes against bacteria into a mutualistic non-inflammatory signaling receptor that fosters close association with bacteria is biologically elegant.

Fuc-TLR4 was not apparent in tissues other than gut epithelium of uncolonized adult mice. However, its presence and activity in the two distinct human cell line models suggests the possibility of fuc-TLR4 inducing surface fucosylation that could alter additional functions in other tissues in response to diverse physiologic stimuli. That a minor change in receptor glycosylation can actuate distinct alternate signaling, resulting in alternate outcomes, goes beyond current emerging evidence of glycosylation altering receptor function. The data herein support a specific function of fuc-TLR4 as a sentinel molecule that induces gut fucosylation in response to the first presence of fucotrophic mutualists in newly mature gut, or the return of fucotrophic mutualists in a poorly colonized or dysbiotic adult gut. This system supports homeostasis of the intestinal mucosa.

Recognition of microbiota by TLRs is required for intestinal homeostasis (13, 16). The mucosal barrier requires glycosylation: distorted glycosylation alters tight junctions, signaling, microbiota composition and fermentation products, and exclusion of pathogens (35). An active *fut2* gene in the mucosa supports colonization by characteristic fucose dependent microbiota in a high-glucose polysaccharide-deficient diet (23). Homeostasis of the intestinal mucosa requires full fucosylation (13, 15). Fecal metabolic products produced by fut2-deficient mammals are distinct from patterns of metabolic products in wild-type animals (23), and these differences coincide with differing intestinal mucosal function. Therefore, the relationship between fuc-TLR4 function, colonization, and homeostasis was investigated. DSS in drinking water induces mucosal injury from which colonized mature mice fully recover. In the absence of colonization and mucosal fucosylation, the intestinal injury becomes more severe and can become lethal (Figure 6). With restitution of mouse microbiota, the mice survive. Recovery of mucosal resilience also followed treatment with UEA1, a more specific fuc-TLR4 ligand than total microbiota (Figure 6B); lectins with other specificities were ineffective in enhancing recovery from colitis. Consistent with these data, neither UEA1 nor recolonization was able to rescue TLR4^−/−^ mice or MyD88^−/−^ mice from the mucosal injury. Thus, fucosylation induced by mutualistic bacteria *via* the fuc-TLR4 sentinel is required to recover from gut injury and restore homeostasis in the intestinal mucosa following antibiotic-induced dysbiosis.

If fuc-TLR4 is essential to colonization, and colonization is essential to health, the absence of fuc-TLR4 or its downstream fucosylation should result in pathology. FX mutant mice, unable to generate GDP-fucose and therefore unable to fucosylate glycans, are normal at birth, but die shortly after weaning from colitis and diarrhea (36). In light of our findings, their inability to fucosylate the mucosa would preclude establishment of a fucose-utilizing mature microbial ecosystem. This would increase opportunity for pathobionts to colonize, and decrease homeostasis and resilience, consistent with the reported bacterial infiltration and inflammatory changes in FX mice. Mucosal fucosylation is also associated with recovery from enteric infection. Mucosal lymphocytes responding to infection induce epithelial fucosylation that can be foraged by protective microbiota (37), offsetting a decline in dietary energy due to inflammatory anorexia. This was purported to decrease the degree of inflammation, increasing host resilience and accelerating recovery (21, 24). The role of mucosal fucosylation in these animal models is concordant with observations in humans.

Strong clinical associations between *FUT2* expression and disease risk (38–42) are consistent with the mucosal fucosylation system being a significant component of immune homeostasis. Approximately 20–25% of human populations of European, Asian, and African descent have homozygous recessive inactive point mutations in their *FUT2* genes, and such “non-secretors” lack appreciable α1,2 fucosylated glycans in exocrine secretions and mucosa (43). The central hypothesis herein predicts that this genetic polymorphism in non-secretors would preclude the health benefit of bidirectional communication between mucosa and fucotrophic microbes through the fuc-TLR4 communication system.

In addition to the glycans expressed on the apical mucosal membrane, early colonization by microbiota can also be influenced by the oligosaccharides in milk. Although all milk contains oligosaccharides, the types, amounts, and timing across lactation differ among species and within individuals of a species, especially between secretor and non-secretor lactating humans. Early human milk of secretors is rich in fucosyloligosaccharides, suggesting a possible role in supporting early colonization of the young gut by fucotrophs, but fucosyloligosaccharides are quite sparse in the milk of non-secretor mothers (44). Hence, in contrast to the mucosa of suckling mice, in humans, the newborn gut of secretors may be fucosylated; accordingly the fucotroph interaction with fuc-TLR4 would be of primary importance in the secretor newborn. A non-secretor infant receiving non-secretor milk from its mother would lack a source of fucose from the milk or from the intestinal mucosa, providing little support to colonization by fucotrophic bacteria.

Genome-wide association studies identify non-secretors as being predisposed to chronic inflammatory conditions involving gut microbiota, including Crohn**’**s disease, primary sclerosing cholangitis, celiac disease, type-1 diabetes, and obesity (38–42). *FUT2* status influences both alpha and beta diversity of fecal microbiota in these populations (23). Moreover, non-secretor premature infants are at significantly higher risk of developing the inflammatory condition necrotizing enterocolitis (NEC) than comparable secretor premature infants (45). The microbiome of secretor and non-secretor neonates differ significantly, and premature infants at highest risk of developing NEC have microbiota whose composition is distinct from early microbiota of those at low risk (45). These associations, in concert with the biological models herein, support *FUT2* expression being central to reciprocal communication between the human intestinal mucosa and human mutualists, thereby fostering mucosal immune homeostasis.

To summarize, this report identifies a novel fucosylated glycoform of TLR4 that has a unique signaling function independent of the NF-κB cascade. Appending an α1,2-fucosylation to the terminus of a preexisting glycan converts TLR4 from an innate immune signaling receptor that mounts pro-inflammatory defensive processes against foreign bacteria into a mutualistic signaling receptor that fosters close association with mutualistic bacteria. In its presence, fucose-utilizing bacteria activate ERK and JNK dependent fut2 transcription and mucosal fucosylation. This fucosylation can support full colonization by a mutualist-dominated microbial community. This interkingdom reciprocal communication is critical to adult colonization in mice, recovery of microbiota devastated by antibiotic treatment, and restoration of homeostasis after intestinal injury. The data herein is, to our knowledge, the first description of how specific fucosylated moieties of TLR4 glycoforms can convert its function from one of exclusion, i.e., signaling inflammation in response to Gram-negative bacteria, to one of inclusion, i.e., inducing a fucosylated niche to promote colonization by Gram-negative mutualists. This mutualist-dominated gut microbiota is associated with recovery from the dysbiosis induced by antibiotic treatment and other pathologic insults, and with restoration of homeostasis of the intestinal mucosa. Defining the etiology and pathobiology of inflammatory diseases associated with mutations of the *FUT2* gene and with dysbiosis may result in novel approaches toward treating some chronic clinical maladies.

## Data availability statement

The original contributions presented in the study are included in the article/supplementary material, further inquiries can be directed to the corresponding author.

## Ethics statement

The animal study was reviewed and approved by IACUC Harvard.

## Author contributions

NN, DM, and DN contributed to experimental design and interpretation of data, and data were obtained by DM and NN. The manuscript was written by DN and NN. All authors contributed to the article and approved the submitted version.

## Funding

This work was supported by National Institutes of Health HD 013021, HD 059140, AI 075663, and HD 059126, and a pilot feasibility grant from the Center for the Study of Inflammatory Bowel Diseases at the Massachusetts General Hospital.

## Conflict of interest

The authors declare that the research was conducted in the absence of any commercial or financial relationships that could be construed as a potential conflict of interest.

## Publisher’s note

All claims expressed in this article are solely those of the authors and do not necessarily represent those of their affiliated organizations, or those of the publisher, the editors and the reviewers. Any product that may be evaluated in this article, or claim that may be made by its manufacturer, is not guaranteed or endorsed by the publisher.
