# Supplementary material for: Fucosylated TLR4 mediates communication between mutualist fucotrophic microbiota and mammalian gut mucosa
Source: Front Med (Lausanne). 2023 Mar 16;10:1070734. doi: 10.3389/fmed.2023.1070734 (PMC10061023; doi:10.3389/fmed.2023.1070734)
Supplement: Supplementary file 1 [file Data_Sheet_1.doc]

**Supplementary material:**


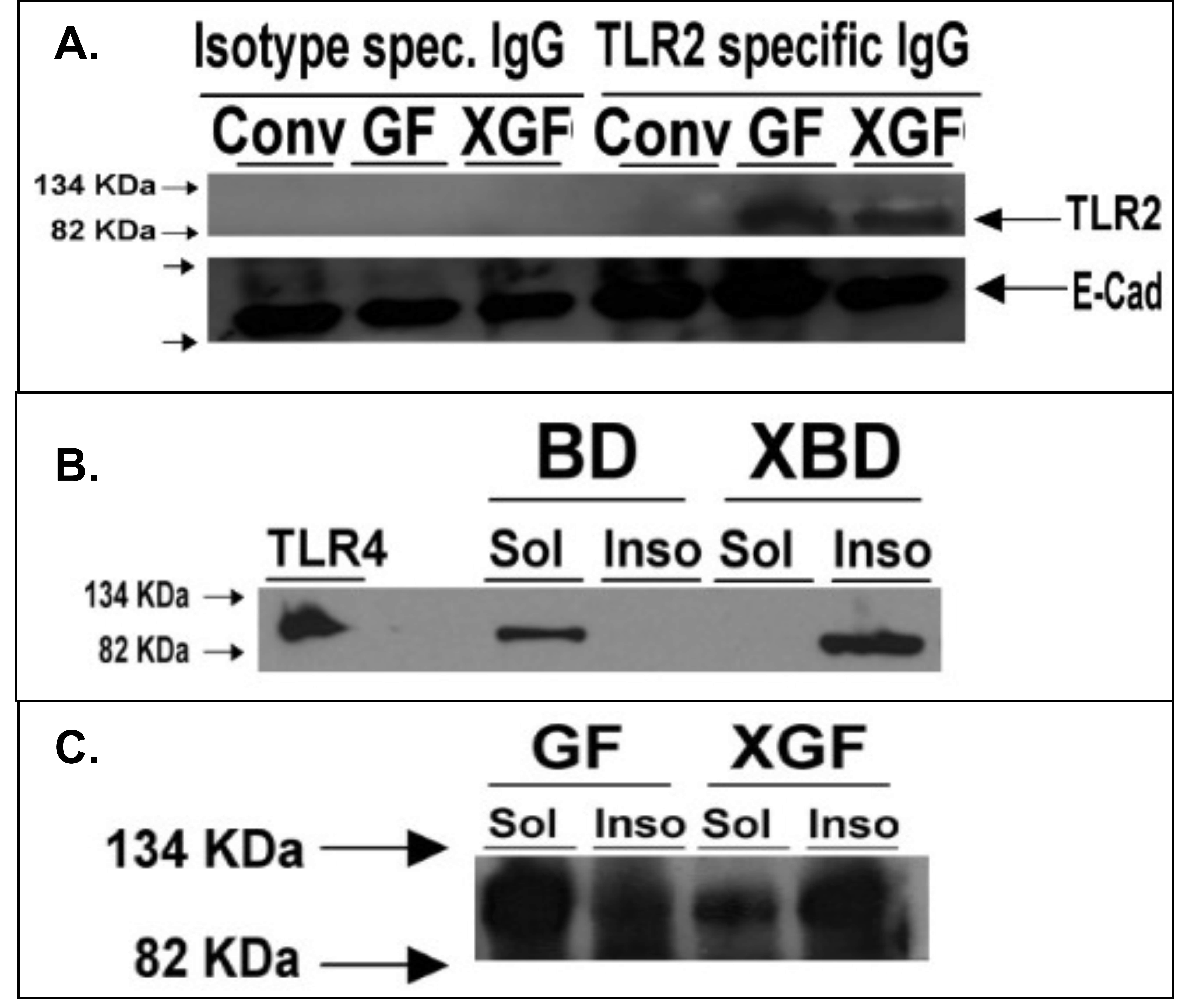


**Supp Figure 1: A.** Control for TLR4 immunoprecipitation from primary colonocytes of conventional (CONV), germ-free (GF) and ex-germ-free (XGF) mice. Control for TLR4 immunoprecipitation (IP) was performed with isotype-matched control IgG and with TLR2-specific IgG; the protein was immobilized and characterized by western blotting analysis (WB). After TLR2 and control immunoprecipitation, E-cadherin protein was immunoprecipitated as a control for total amount of protein used for immunoprecipitation from CONV, GF, and XGF mice. **B.** The soluble (Sol) and insoluble (Inso) membrane proteins from the primary colonocytes of conventional (CONV), bacteria-depleted (BD) and ex-bacteria-depleted (XBD) mice were isolated, and TLR4 protein was detected by western blot analysis. A positive control for TLR4 was fractionated in the first lane.


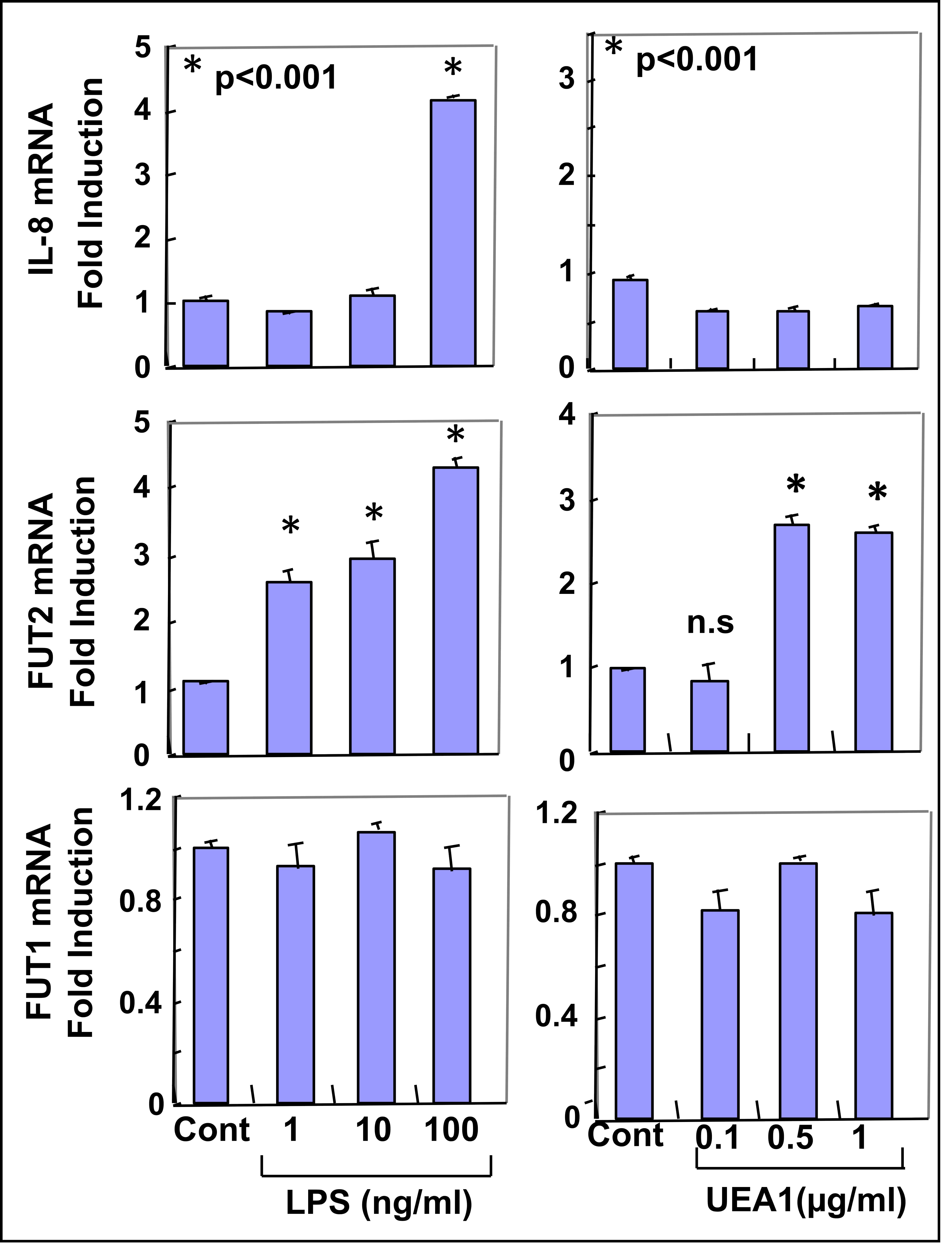


**Supp Figure 2:** Comparison of the ability of LPS and UEA1 ligands to activate fucosylated-TLR4 in Hel cells. LPS was able to induce a classical inflammatory response as measured by IL-8 release mediated through the NF-κB signaling pathway. In contrast, UEA I, which does not stimulate the NF-κB pathway, does not induce IL-8 (upper panels). However, *FUT2* expression can be induced by both ligands of fucosylated TLR4 (middle panels). Neither of these ligands alters the level of *FUT1* mRNA expression (bottom panels). Also, the level of *FUT1* mRNA expression is unaltered by colonization of the colon. These data indicate that binding to a fucose-containing moiety, such as that found on fucosylated-TLR4, allows induction of non-inflammatory genes such as *FUT2* mRNA without stimulation of the NF-κB-dependent pro-inflammatory pathway. In contrast, stimulation by LPS, which occurs independently of fucosylation, was able to induce release of both the inflammatory chemokine, IL8 and the non-inflammatory *FUT2* mRNA.


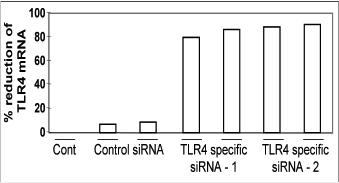


**Supp Figure 3:** In Hel cells, the level of TLR4 expression was significantly abolished by TLR4 specific siRNA, but not by a scrambled negative control siRNA. The level of TLR4 mRNA was measured by qRT-PCR after treatment with two different TLR4 specific siRNAs, performed in duplicate.


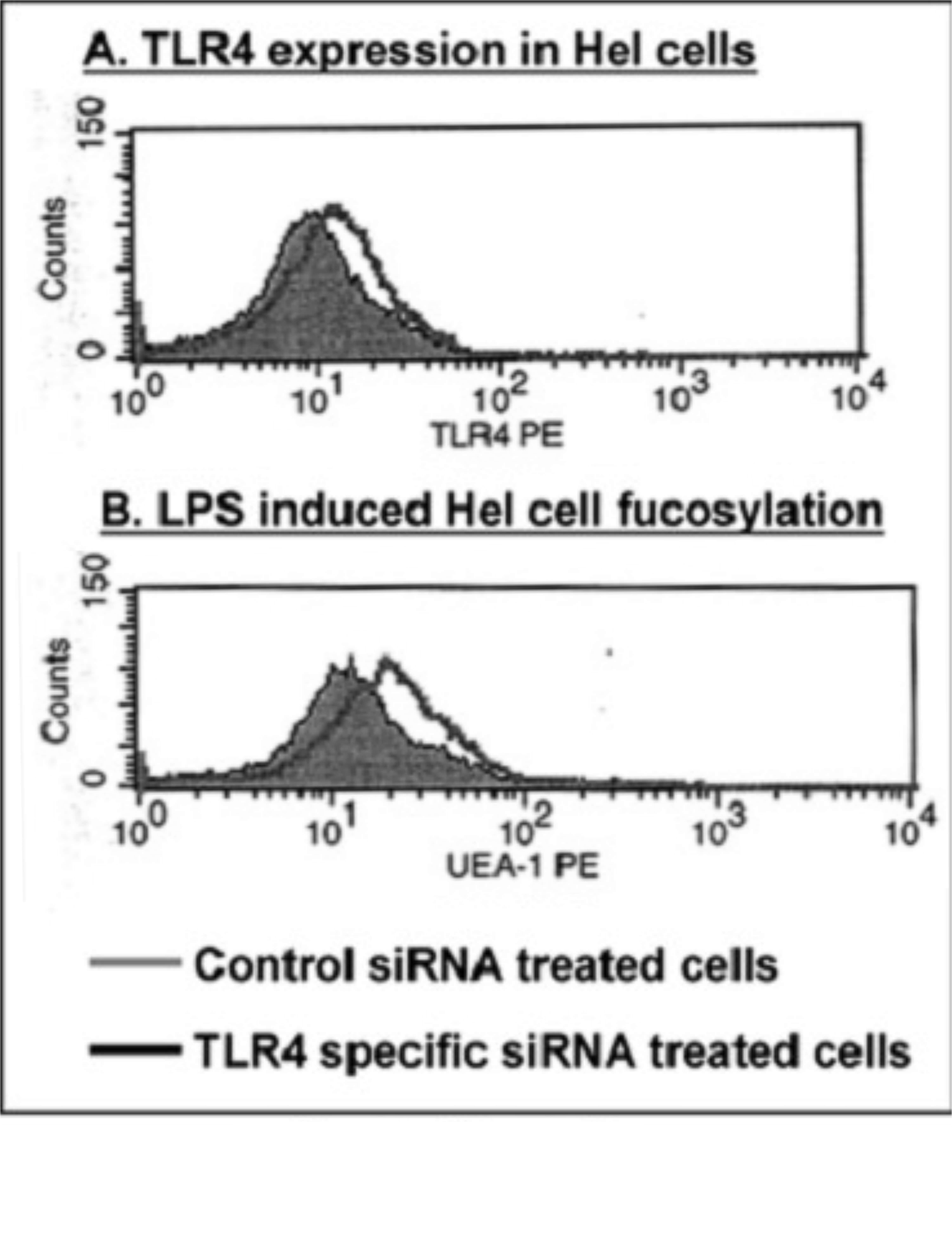


**Supp Figure 4:**  **A.** The cell surface expression of TLR4 in Hel cells was measured by FACS analysis. The cells were treated with TLR4 specific siRNA or a scrambled negative control siRNA. The cell surface expression was measured by mouse monoclonal antibody specific for human TLR4. **B.** After Hel cells were treated with TLR4 specific siRNA or the scrambled negative control siRNA, cells were stimulated with LPS and cell surface fucosylation was measured by FACS analysis as binding to UEA I. The induction of cell surface fucosylation by LPS was abolished by treatment with TLR4 specific siRNA but not with the scrambled negative control siRNA.
